# The science of uncertainty guides fetal-neonatal neurology principles and practice: diagnostic-prognostic opportunities and challenges

**DOI:** 10.3389/fneur.2024.1335933

**Published:** 2024-01-30

**Authors:** Mark Steven Scher

**Affiliations:** Fetal/Neonatal Neurology Program, Division of Pediatric Neurology, Department of Pediatrics, Case Western Reserve University, Cleveland, OH, United States

**Keywords:** fetal-neonatal neurology, neural exposome, gene-environment interactions, maternal-placental-fetal triad, social determinants of health, science of uncertainty, shared clinical decisions prevailing family values diagnostic, therapeutic

## Abstract

Fetal-neonatal neurologists (FNNs) consider diagnostic, therapeutic, and prognostic decisions strengthened by interdisciplinary collaborations. Bio-social perspectives of the woman’s health influence evaluations of maternal-placental-fetal (MPF) triad, neonate, and child. A dual cognitive process integrates “fast thinking-slow thinking” to reach shared decisions that minimize bias and maintain trust. Assessing the science of uncertainty with uncertainties in science improves diagnostic choices across the developmental-aging continuum. Three case vignettes highlight challenges that illustrate this approach. The first maternal-fetal dyad involved a woman who had been recommended to terminate her pregnancy based on an incorrect diagnosis of an encephalocele. A meningocele was subsequently identified when she sought a second opinion with normal outcome for her child. The second vignette involved two pregnancies during which fetal cardiac rhabdomyoma was identified, suggesting tuberous sclerosis complex (TSC). One woman sought an out-of-state termination without confirmation using fetal brain MRI or postmortem examination. The second woman requested pregnancy care with postnatal evaluations. Her adult child experiences challenges associated with TSC sequelae. The third vignette involved a prenatal diagnosis of an open neural tube defect with arthrogryposis multiplex congenita. The family requested prenatal surgical closure of the defect at another institution at their personal expense despite receiving a grave prognosis. The subsequent Management of Myelomeningocele Study (MOMS) would not have recommended this procedure. Their adult child requires medical care for global developmental delay, intractable epilepsy, and autism. These three evaluations involved uncertainties requiring shared clinical decisions among all stakeholders. Falsely negative or misleading positive interpretation of results reduced chances for optimal outcomes. FNN diagnostic skills require an understanding of dynamic gene-environment interactions affecting reproductive followed by pregnancy exposomes that influence the MPF triad health with fetal neuroplasticity consequences. Toxic stressor interplay can impair the neural exposome, expressed as anomalous and/or destructive fetal brain lesions. Functional improvements or permanent sequelae may be expressed across the lifespan. Equitable and compassionate healthcare for women and families require shared decisions that preserve pregnancy health, guided by person-specific racial-ethnic, religious, and bio-social perspectives. Applying developmental origins theory to neurologic principles and practice supports a brain health capital strategy for all persons across each generation.

## Introduction: the importance of shared clinical decisions in fetal-neonatal neurology

Fetal-neonatal neurologist (FNN) training provides opportunities for the healthcare provider to improve career-long learning that sustains accurate and ethically sound clinical decisions across the person’s lifespan (1). Current levels of reproductive and pregnancy healthcare have knowledge gaps that challenge this diagnostic process. Specificity and sensitivity limitations of current prenatal surveillance studies can result in falsely negative or positive interpretations. Application of developmental neuroscience principles with each clinical encounter can help maintain accuracy and avoid biased thinking. Diagnostic uncertainties to predict brain health or disease present challenges from childhood through adulthood. Three clinical scenarios initiated as prenatal FNN consultations are presented that stress the importance of integrated clinical and bio-social perspectives. Shared clinical decisions regarding brain health or disease diagnosis and prognosis must be reconsidered as new phenotypes appear across the developmental-aging continuum. Efforts to maintain diagnostic accuracy acceptable to all stakeholders will help sustain a productive social contract with trust (2). All persons require equitable and morally grounded healthcare (3). This commitment to high standards for clinical practice must be offered across a person’s lifespan. These principles will guide future neurologic research that supports a brain health capital strategy that benefits each and successive generations (4).

### Misdiagnosis of a meningocele as an encephalocele

A primigravid 24 years-old white woman presented to an outside obstetrical facility in 2000 to confirm her suspected pregnancy. At 10 weeks gestation, a sonogram documented a presumed occipital encephalocele. As a professional journalist, she had sufficient training to acquire medical information through internet research regarding her child’s diagnosis and prognosis, assuming a presumed ventral neural tube defect. She and her partner had no known inherited genetic diseases and experienced no childhood or adult medical risks or illnesses. No aneuploidy was documented on karyotypic screening with normal serologies for congenital infections without evidence of active infections. A normal alpha fetoprotein test (AFP) result had been obtained by her obstetrician, suggesting a closed form of spina bifida. Mother was referred to a maternal-fetal medicine service at her health system at which time this anomaly was again suggested. No fetal brain MRI was pursued to confirm this provisional diagnosis of an encephalocele. Her physicians supported by a genetic counseling consultation advised termination of her pregnancy.

Legislative restrictions in her state in 2000 limited elective termination to less than gestational week 24. An urgent second opinion at our pediatric-obstetrical center was requested by mother at gestational week 12. An interdisciplinary evaluation by the maternal-fetal medicine service expedited coordinated pediatric neurology, neurosurgery, and genetic consultations. Medical records of mother’s previous prenatal care were reviewed, and a second normal AFP test was obtained. Repeat sonographic imaging documented an occipital mass that suggested a meningocele rather than an encephalocele. A 13 weeks brain MRI (1 magnification Tesla using current HASTE technology) confirmed an extra-cerebral cystic mass consistent with an occipital meningocele without discernable cerebral dysgenesis. A sonographic anatomical survey also documented no other organ-specific anomalies. Diagnostic limitations based on current MRI technology were explained to mother that could not eliminate possible anomalous brain development below the resolution of fetal brain MRI images (5). Diagnostic accuracy using 4D sonography with present day improved brain MRI Tesla resolution now more often offers more definitive structural descriptions for more accurate interpretations (6). Pregnancy termination consequently was not advised. A more favorable outcome including possible normal neurodevelopment was suggested, assuming no further pregnancy complications (6).

Mother subsequently presented during her third trimester of pregnancy with rapidly worsening preeclampsia at gestational week 32. She required immediate hospitalization and treatment for suspected eclampsia. Early delivery was advised after consultation with the neonatology service, anticipating complications of prematurity that would require neonatal intensive care. After one dose of antenatal corticosteroids was administered, an urgent cesarean section was chosen given mother’s clinical status and anticipated complications of a vaginal delivery given this brain anomaly. Rupture of the meningocele occurred during delivery. Pediatric neurosurgery performed surgical closure after postnatal brain MRI confirmed no discernible cerebral dysgenesis including hydrocephalus.

The child required ventilatory assistance including inhaled nitric oxide despite surfactant treatments, based on current practice guidelines (7). Placental examination documented histological malperfusion lesions on both maternal and fetal surfaces. She experienced systemic complications of prematurity including a prolonged need for respiratory care for chronic cardiopulmonary disease, systemic dysautonomia with apneas and bradycardias requiring pressors and caffeine, and slow weight gain requiring prolonged gavage feeding. The child gradually exhibited improved state regulation over a 3 weeks hospital stay, assisted by developmental care by the nursing staff assisted by the family. No sepsis occurred, and unilateral grade 2 intraventricular hemorrhage without progression on serial ultrasound studies was documented. Mother initially required intensive care management to control her acute hypertensive episode with complete resolution without the need for medication by her discharge.

The child required close neurodevelopmental follow-up following discharge consisting of multiple therapy interventions and gastric feeding with oral supplements with slow gradual weight gain. During her first 18 months of life, she exhibited delayed motor and language milestones despite normal hearing and vision. Pediatric neurosurgery monitored her neurologic progress until 2 years of age including a 12 months brain MRI that documented normal structures. The family’s pediatric primary care practice, with pediatric neurology and developmental-behavioral pediatric consultations, was coordinated with an early intervention program. The child eventually reached age-appropriate four-domain developmental milestones by her third birthday. She entered preschool with an individual educational plan anticipating the need for cognitive and behavioral interventions supported by her parents’ understanding and participation. She subsequently demonstrated sufficient improvements to justify discontinuation of supplemental educational and therapy services by 6 years of age. Pediatric neurology consultations were discontinued at 10 years of age given no active neurologic or mental health concerns. Later contact with her mother indicated that her daughter completed high school and college and was enrolled in postgraduate education.

### Two maternal-fetal dyad consultations with fetal cardiac rhabdomyoma

FNN and genetics consultations were requested by the maternal-fetal medicine service at our obstetrical-pediatric center for two different pregnancies regarding multiple fetal cardiac rhabdomyomas. First trimester sonography identified these lesions with diagnostic input from the pediatric cardiology service. Each consultation occurred within the same calendar year (2001). A multi-organ fetal sonographic survey performed at gestational week 18 documented no anomalies for either fetus. No outflow obstruction or hydrops fetalis developed for either fetus. Discussions with each mother, her partner, and extended families centered on the provisional diagnosis of tuberous sclerosis complex (TSC) based on published associations of this genetic disorder given multiple cardiac rhabdomyoma despite no other organ anomalies (8, 9). Neither mother had known family members with genetic disease nor had experienced medical conditions suggestive of TSC. Detailed evaluations of each set of parents by the pediatric geneticist documented no examination findings or historical evidence of systemic effects of this autosomal dominant genetic condition. No prenatal TSC carrier testing for a TSC 1 or 2 exon coding mutations was clinically available at the time of their medical care. Postnatal investigations were discussed to more definitively reach or reject this genetic diagnosis based on inferential evidence including neuroimaging despite the absence of reliable genetic testing.

One mother was white with a college education who conducted her own independent internet review of TSC and concluded that her child had a grave prognosis. Despite coordinated consultations with recommendations from the MFM, genetics, and neurology services, mother chose to travel out of state to terminate her pregnancy. She relied on her own conclusions despite her healthcare providers’ stated uncertainties pending further evaluation. An urgent elective abortion was performed at an obstetrical center in an adjoining state, given that her state of residence had legislative limitations to perform this procedure beyond gestational age of 23 weeks. No fetal brain MRI or postmortem evaluations were obtained prior to her pregnancy termination.

The second mother was black with blue-collar employment after finishing high school and 2 years of college. Mother attended all prenatal care visits with her partner and selected family members who participated in lengthy discussions with the providers. Mother shared with her healthcare providers that she understood and was prepared for the possible grave prognostic consequences associated with confirmation of TSC. Mother made a valued informed decision based primarily on her religious beliefs with the support of her family to continue with prenatal care. She and her partner explained that they were prepared to provide care for her son’s long-term sequelae even if he was ultimately diagnosed with TSC. Financial assistance by her family was discussed to travel out of state for a pregnancy termination. However, mother and her partner rejected this option. Mother gave birth to her son who later had clinical and neuroimaging confirmation of TSC in the absence of diagnostic evidence of this genetic disorder for either parent. The child experienced severe developmental delay, autistic spectrum disorder, and intractable epilepsy into early adulthood. Multidisciplinary healthcare was coordinated with his parents’ participation, including rehabilitative services in and outside the school setting. He has been challenged with daily seizures as well as cognitive and social-adaptive challenges. His complex medical condition was addressed by parents and family through adherence to serial healthcare recommendations and IEP adjustments at school, supplemented by supplemental therapies out of school. Their adult son remains at home with the family after completing a special educational program at school through 22 years of age. He continues to be included in all family social and religious activities. Enrollment in a community-based residential daytime program provides limited supervised employment opportunities adjusted to his abilities. Multiple hospitalizations were required during childhood and adulthood to address acute concerns, primarily related to acute exacerbation of his condition of intractable epilepsy without multisystemic complications.

### An open neural tube defect with arthrogryposis multiplex congenita

A primigravid 24 years-old woman was referred for pediatric neurologic and neurosurgery consultations by the maternal-fetal medicine and genetics services after the detection of an open neural tube defect (NTD) involving L-1 through S-5 levels with no discernable ventriculomegaly. This was based on sonographic visualization during the 15 week of pregnancy in 1999. Their unborn son was also noted to have multiple proximal joint contractures consistent with diagnosis of arthrogryposis multiplex congenital (AMC). No multi-organ anomalies supported fetal akinesia syndrome (10). There was no documented hydrops with a visualized stomach size and volume. A high risk of lethality or significant neurodevelopmental delay with survival was shared with the woman and her partner (11, 12). Routine karyotypic and serologic screening during her first trimester surveillance documented no abnormalities. Parental family histories were negative for brain anomalies. Mother and her partner were informed of the grave prognostic implications for their child given the extensive open spinal defect in the context of a possible genetic disorder based on AMC. A cesarean section was advised followed by an emergent postnatal neurosurgical closure of the open NTD. Risks for shunt-dependent hydrocephalus and long-term multidisciplinary care were explained that would more likely involve serial medical and surgical evaluations given anticipated complications. The presence of AMC associated with developmental delay raised suggestions for a diagnostic evaluation for a genetic disorder expressed with severe developmental disorders. His probable need for continuous individual educational and therapy services through his school years was also shared with the family. They were encouraged to seek guidance and maintain partnership with pediatric healthcare services to address the medical and surgical challenges associated with his complex diagnosis. These diagnostic, therapeutic, and prognostic recommendations had been offered prior to the published findings offered by the Management of Meningomyelocele Study (MOMS) (13).

After receiving our prenatal consultations suggesting that their child would experience grave neurodevelopmental deficits, the family sought a second opinion at another institution where prenatal surgical closure of the defect was being performed for similar fetal patients. Parents independently sought this second opinion based on an internet advertisement from this institution reporting possible positive results. More successful outcomes with less risk for shunt-dependent hydrocephalus and improved ambulation were reported by the interdisciplinary medical team at this institution for those children who received this fetal surgical procedure. These claims were offered prior to the agreed moratorium among medical specialties to organize and conduct MOMS. Later published recommendations would have judged this child to be ineligible for the fetal surgical procedure given his risks for genetic disease in the context of an extensive open spina bifida with AMC (14). Given the documented AMC at our institution, a concern had been shared with the parents before her decision to request for this procedure, assuming an association with a genetic disorder with worse prognosis (15).

The procedure was performed at the family’s personal expense without insurance coverage. Mother returned to our obstetrical medical center for maternal care. Despite receiving this fetal repair, this child was born prematurely at gestational age of 32 weeks. He required 6 weeks of acute and step-down neonatal intensive care for multiple complications associated with prematurity. This included prolonged ventilatory care and extensive orthopedic and therapy evaluations. Serial surgical procedures were anticipated to mitigate the extent of musculoskeletal complications from AMC.

Interdisciplinary healthcare was coordinated after discharge with the family’s chosen primary pediatric service to address the multisystemic complications of a neural tube defect. Rehabilitative services coordinated with subspecialty medical and surgical interventions were required. A ventriculoperitoneal shunt was placed to treat obstructive hydrocephalus with the need for one revision. He failed to achieve limited ambulation and required urologic interventions with a catheterization program. A comprehensive individual educational plan during his school years with parental and professional interactions was established. Psychometric scores consistent with severe intellectual disability and autistic spectrum disorder were documented that guided changes to his biannual educational plans. His parents chose not to accept a pediatric genetic specialty referral and stated their decision not to conceive other children. The couple eventually divorced but continued to live in the same community, working together to advocate for their child’s ongoing medical needs into adulthood. He lives with his mother and receives outpatient residential supervised instruction as well as medical services for medical complications given his severe global developmental delay and intractable epilepsy.

## Maternal levels of care offer diagnoses with recognized limitations

FNN consultants require the most accurate medical information for each MPF triad consultation, integrated with knowledge of social determinants of health and bioethical principles. Understanding of developmental neuroscience helps meet challenges during the first 1,000 days. However, present knowledge gaps reduce diagnostic accuracy. Reproductive and pregnancy healthcare information among all stakeholders can help formulate accurate though provisional prospective decisions, requiring reevaluations given changes in the child’s clinical status relative to illnesses and complications across advancing ages. Prenatal prognostic assessments are formulated based on the presence, extent, and specificity of fetal brain structural and functions deficits after evaluation of the maternal-placental-fetal (MPF) triad. A long interval often separates prenatal disease pathways with later phenotypic expressions during childhood and adulthood (16, 17). This diagnostic uncertainty requires multiple shared clinical decisions with the woman and family regarding their child’s outcome. Providers must also consider possible functional improvements reflecting neurodiversity with maturation despite varying degrees of permanent sequelae. Multiple challenges contribute to diagnostic errors based on the interpretation of current fetal surveillance testing. These include limited sensitivity and specificity to identify the disease, mother’s barriers to adequate healthcare, her lack of compliance with medical advice based on cognitive limitations and implicit biases, and providers’ perceived attitudes and errors of omission and commission.

Routine levels of maternal care are established with adjustments throughout pregnancy based on serial assessments of medical findings that influence risk estimation. This stratification of prenatal care needs to consider mother’s access to timely medical care and her health literacy. Possible mental health challenges will amplify the stressful experiences by the woman during her high-risk pregnancy, affecting compliance with providers’ recommendations (18–20). Detailed family histories together with mother’s reproductive history help anticipate periconceptional and pregnancy medical conditions that increase disease risks to the mother and her child. Multiple diseases and adversities experienced by the woman carry increased reproductive risks. More prevalent examples include hypertensive disorders (21), diabetes mellitus (22), obesity (23), autoimmune conditions (24), and infectious illnesses (25). Multiple gestation pregnancies and a history of infertility requiring assisted reproductive technologies necessitate higher levels of pregnancy surveillance. These conditions when effectively managed can contribute to more favorable outcomes. However, increased risks for adverse maternal and fetal outcomes also may occur given confounding risk factors such as zygosity, placental disease, maternal diseases, prematurity, and associated genetic disorders (26–29). Advanced maternal age (<35 years) (30), previous spontaneous miscarriage and stillborn (31), and familial or complex medical disease conditions associated with familial backgrounds (32, 33) similarly require greater prenatal medical surveillance to avoid or reduce the incidence of adverse maternal and fetal outcomes. Social determinants of healthcare need to be recognized to avoid inequities before and during each pregnancy (34). Medical deserts in resource-challenged communities (35) create barriers to healthcare for women during and between pregnancies (36) which contribute to adverse effects on their children (37).

**Figure 1:**
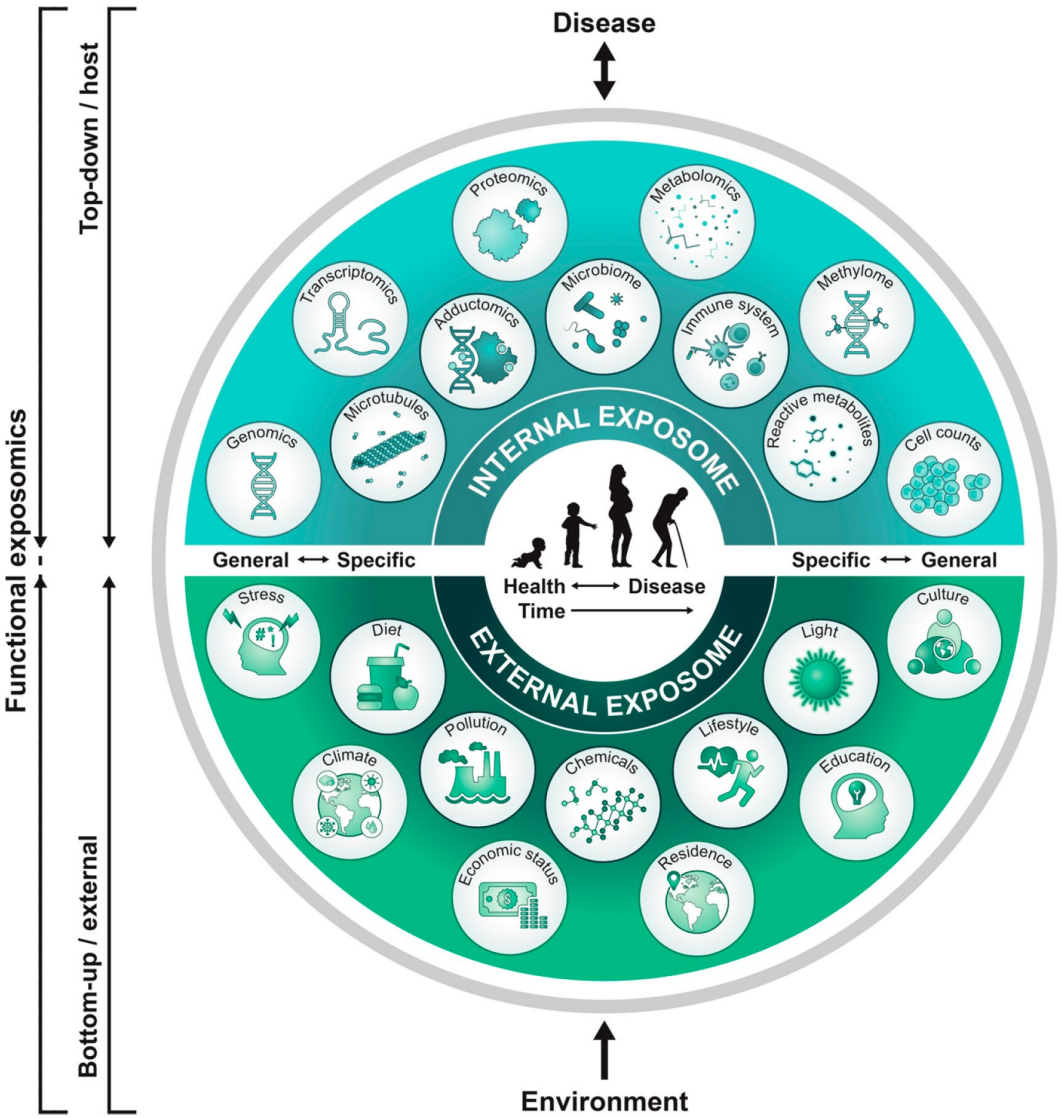
Functional approach to study the exposome across the lifespan: A top-down approach uses molecular epidemiology studies focused on biologic response profiles with xenobiotic exposures using “omics” technologies on host biospecimens. This approach generates hypotheses regarding exposure-disease and exposure-response relationships without necessarily capturing direct measures of exposure. Applying the bottom-up approach, comprehensive data on environmental exposures are collected through surveys, sensors, or trace chemical analyses of biospecimens to generate hypotheses on effects without necessarily investigating a specific effect. A functional exposomics study bridges these two approaches, comprised of the biologically active exposures to the individual and with specific evaluations to assess associations between environmental exposures and biological effects as a function of advancing age (81).

The exposome research field was introduced nearly two decades ago (38) suggesting that gene and environment (G × E) interactions can more accurately model healthcare applied to cancer research. Interdisciplinary applications of this concept now encompass the reproductive and pregnancy exposomes which influence the maternal-placental-fetal triad, neonate, child, and adult. Synergistic reproductive, pregnancy, and pediatric exposome effects impact the neural exposome with phenotypic expressions of brain health or disease across advancing ages (39). Endogenous and exogenous toxic stressor interplay (TSI) represent biological-chemical interactions involving internal and external exposomes that contribute to neurologic disorders expressed during one or successive lifespans (40) (Figure 1).

The FNN can leverage the principles of developmental neuroplasticity that influences the neural exposome to improve clinical practice accuracy and continuity of care. Enhanced diagnostic and prognostic skills acquired with formal training can more accurately recognize and distinguish normal expressions of neurodiversity from permanent brain disorders during clinical practice. Interdisciplinary neuropalliative programs have more recently presented successful strategies for integrated pediatric care beginning during neonatal life with applications of prenatal information based on fetal neurology consultations (41). The following sections discuss present and future strategies that can be employed by the FNN to improve shared decision-making. Errors of omission and commission must be recognized and avoided when offering shared clinical decisions. Relevant facts from the chosen case vignettes are subsequently reemphasized that introduce a proposed brain health capital strategy (42) applicable for all stakeholders.

## Traditional fetal neurology consultations rely on sonographic findings

Fetal neurology consultations have traditionally been requested to provide clinical correlations for structural abnormalities documented by abdominal sonography. These evaluations may be initiated after the results are reported during untargeted maternal screening studies at scheduled gestational age ranges. Abnormalities may also be shared with the FNN consultant during participation in outpatient maternal care, maternal imaging procedures, or with high-risk interdisciplinary MFM evaluations. Abnormalities in structural fetal brain maturation consist of anomalous and destructive lesions. Greater severity of lesions may be better visualized as MPF triad diseases (43) worsen across three trimesters of pregnancy. Referrals are often initiated by the obstetrical or maternal-fetal medicine services following the anatomical survey of the fetus between 18- and 24 weeks gestational age when greater sonographic imaging details detect brain lesions. Fetal cardiologists currently can receive additional training with certification to perform independent evaluations to more accurately apply their diagnostic perspectives based on their subspecialty training (44). Fetal neurologists presently are not offered similar training. Active participation in the diagnostic process by the FNN during studies interpreted by the obstetrical team in the maternal imaging suite can help compensate for this lack of formal instruction. Formal sonographic certification would be advantageous.

Brain anomalies are uncommon findings when compared with all prenatal anomalies. Conventional fetal sonographic surveillance followed by fetal MRI confirmation has identified brain anomalies in between 0.1 and 0.2% of live births (45). Given this is the principal reason for a FNN consultation, there remains an implicit bias among obstetrical and interdisciplinary pediatric subspecialties that assumes that pediatric neurologists lack sufficient knowledge and experience to consider a wider range of more prevalent MPF triad diseases associated with fetal brain diseases. A past survey of pediatric subspecialists has in fact ranked pediatric neurologists as the least likely to have received formal fetal neurology training and were the least prepared to participate in prenatal pediatric evaluations (46).

One fetal neurology service two decades ago reported findings from a retrospective review consisting of evaluations, based on a more comprehensive FNN program. An expanded FNN consultative role by a single pediatric neurologist was offered during prenatal care, coordinated through a combined maternal-pediatric health center that requested either formal interdisciplinary consultations or was offered input during fetal imaging procedures or interdisciplinary maternal-fetal medicine conferences. Over a 5 years period, fetal and neonatal brain anomalies were reported based on sonography for approximately 50% of 177 identified maternal-fetal dyad at this tertiary maternal-pediatric health center. Primary anomalous brain lesions included neural tube defects, holoprosencephaly, and schizencephaly (47). The remaining 50% of case histories offered fetal neurologic opinions involving non-CNS systemic-specific anomalies that were identified with suspected effects on fetal brain development. In decreasing order of occurrence, cardiac, renal, gastrointestinal, pulmonary, fetal growth restriction, and hydrops fetalis were described with or without active diseases detected by assessments of the MPF triad. These conditions were discussed with the FNN at interdisciplinary MFM conferences, during procedures in the maternal imaging suite, or when women were hospitalized for acute medical concerns. Evidence-based published literature was considered by the FNN when discussing etiopathogenetic pathways for a particular MPF triad with potential contributions to fetal brain disorders. Maternal illnesses were principally represented by hypertensive disorders, infectious-inflammatory states, and gestational diabetes mellitus representing system-specific illnesses. Pathological mechanisms affecting the fetal brain were considered even without discernible sonographic abnormalities or only suggested by non-specific brain anomalies such as mild ventriculomegaly or midline brain dysgenesis, as represented by an absent septum pellucidum. Specific types and combinations of fetal brain lesions were later associated with some children who expressed functional neurodiversity with normal outcome contrasted with those who experienced varying degrees of more significant neurologic disorders. Future outcome studies require study methodologies that can more accurately select study variables and identify confounders that can more accurately identify time-dependent G × E interactions that impair the neural exposome with neurologic disorders presenting across the developmental-aging continuum.

Despite this earlier published experience advocating for expanded FNN participation, two currently active FNN programs continue to stress limited fetal neurology training guided primarily by referrals based on sonographic documentation of brain lesions (48, 49). Proposed neurology curricula nonetheless are more recently presented that stress greater attention to prevalent pregnancy-related disorders that adversely influence life course brain health even without sonographic findings (1, 39). Combined maternal-pediatric hospital programs can more comprehensively prepare a FNN trainee for career-long learning with supervised consultations that rely on a more comprehensive range of MPF triad diseases that potentially contribute to neurologic disorders. Developmental origins of health or disease and life course theories support this curriculum approach to better anticipate brain diseases expressed across the lifespan (50).

Large brain malformations are more likely to be documented during the first half of pregnancy as exemplified by the chosen case vignettes in this review. The FNN must consider abnormal neurogenesis, differentiation, migration, apoptosis, axonal connections, and synaptogenesis (51). Lesions may have been established with fertilization through inherited or *de novo* genetic disease pathways. Examples of anomalies include neural tube defects (dorsal induction), holoprosencephaly (ventral induction), microcephaly or megalencephaly (neuronal proliferation and apoptosis), lissencephaly, cobblestone malformation, or heterotopia (neuronal migration), and polymicrogyria or cortical dysplasia (cortical organization). Post-translational dysregulation also contributes to brain lesions through diverse cellular and molecular pathways based on mechanisms involving mosaicism (52, 53), imprinting (54), and multiple forms of epigenetic processes (55).

Focal cortical dysplasia exemplifies one malformation of cortical development associated with intractable epilepsy (56). These lesions are often not visible on fetal neuroimaging, even with interpretations combining sonographic and brain MRI findings. Surgical excisions coordinated by comprehensive epilepsy programs for children or adults offer interventions that attempt to offer improved seizure control. Presurgical cognitive-behavioral deficits of many candidates often reduce chances for favorable outcomes despite surgical removal of diseased brain tissue. Recent neuropathological descriptions of the ablated cortical tissue samples from these children and adults documented immunopathological biomarkers of impaired neurogenesis during the first half of pregnancy (57). The chosen synaptic biomarkers in this study represented early excitatory-inhibitory imbalance that later impaired fetal brain connectivity and contributed to epileptogenesis. Seizure expression often begins for the child before 2 years of age (58). Diverse neuronal progenitor populations during embryonic and early fetal brain development preceded dysfunctional neural network development during the latter half of pregnancy. Postnatal ictogenesis was often triggered during communicable or non-communicable disease states, beginning during neonatal life. Impaired interneurons, glia, and microglial populations during early pregnancy, with later altered interconnections, contribute to a wide range of neurologic sequelae across the lifespan including epilepsy.

**Figure 2:**
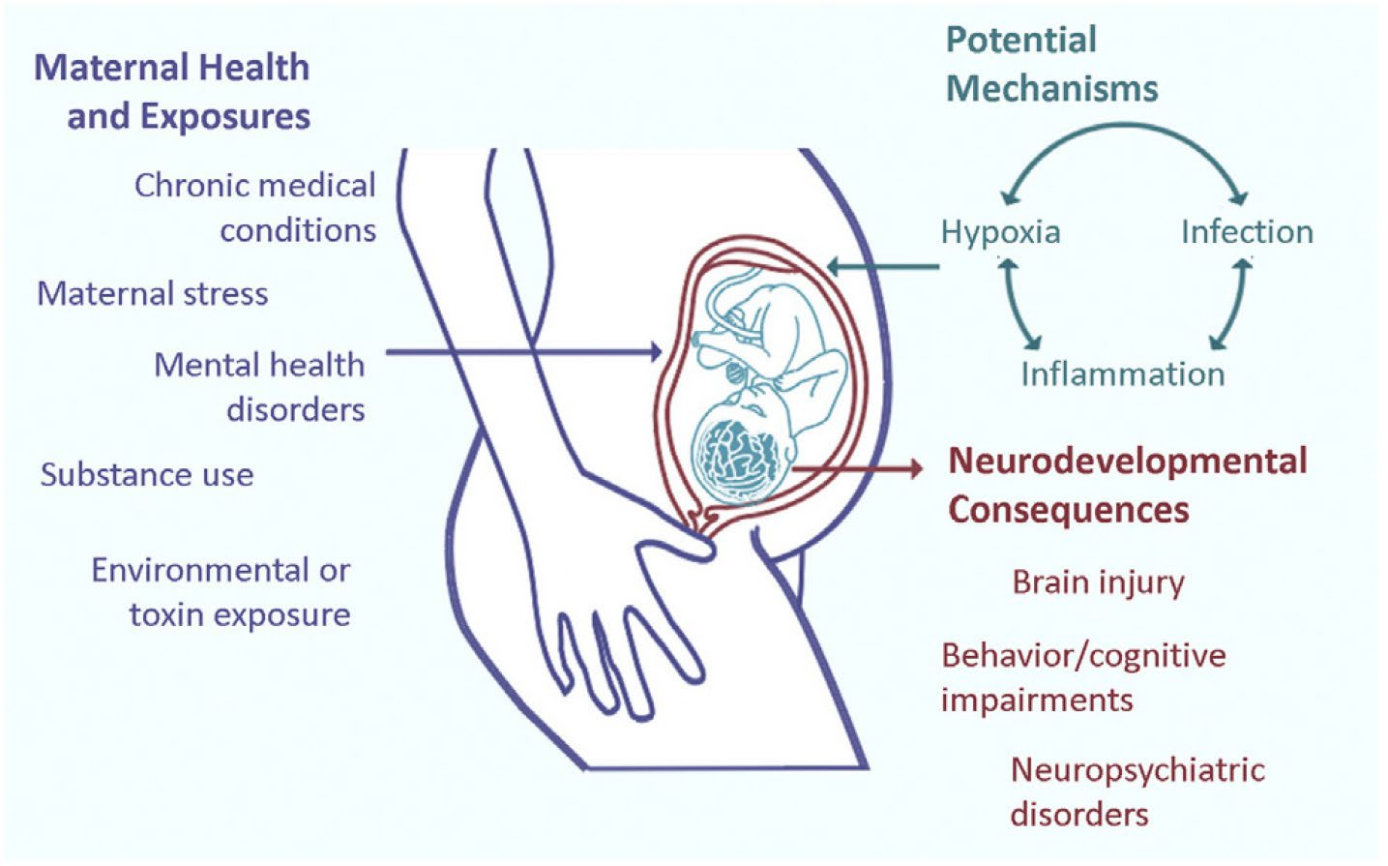
Prenatal adverse exposures to the maternal-placental-fetal triad such as maternal stress, drug use, exposure to environmental toxins, and hypoxia, among other disease pathways more likely collectively influence long-term neurologic structure and function. Current research suggests that three prominent placental disease mechanisms, maternal immune activation, ischemic placental syndrome, and the fetal inflammatory response mediate the relationships between exposures and effects on the fetal brain with long-term abnormal neurologic outcomes (61).

**Figure 3:**
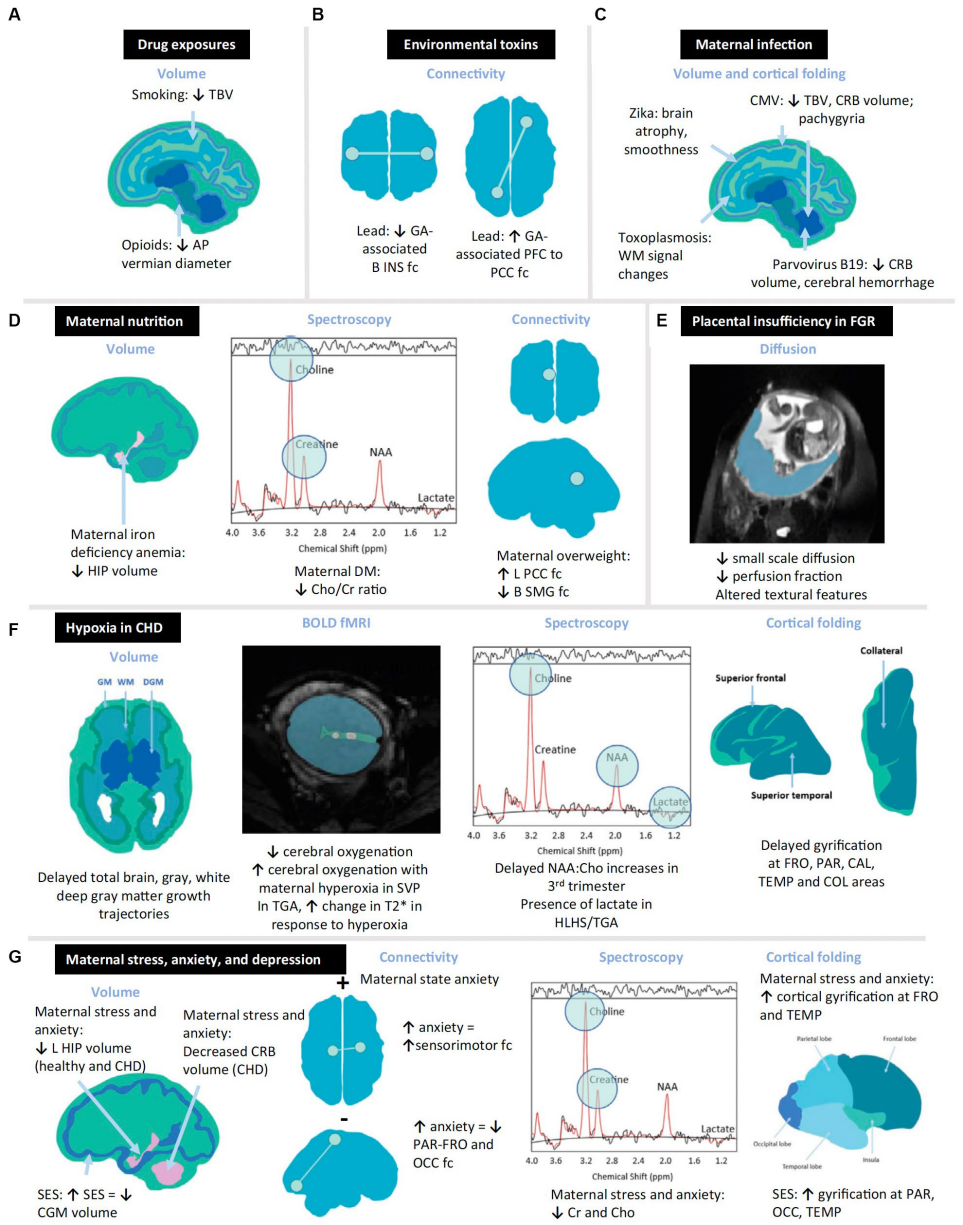
Schematic diagram summarizing structural and functional changes in the *in vivo* fetal brain associated with prenatal exposures. Quantitative neuroimaging modalities depicted utilize volumetric, spectroscopic, and connectivity modalities to depict altered fetal brain. Examples of toxic stressor interplay based on multiple research studies are cited in this review (61). Examples include the following: **(A)** drugs, **(B)** environmental toxins, **(C)** maternal infection, **(D)** maternal overweight or malnutrition, **(E)** placental insufficiency, **(F)** hypoxia, and **(G)** maternal stress. AP, anteroposterior; B, bilateral; BOLD, blood oxygen level-dependent; CAL, calcarine; CGM, cortical gray matter; CHD, congenital. Heart disease; Cho, choline; Cr, creatine; CMV, cytomegalovirus; COL, collateral; CRB, cerebellum; DGM, deep gray matter; DM, diabetes mellitus; fc, functional connectivity; FGR, fetal growth restriction; fMRI, functional magnetic resonance imaging; FRO, frontal; GA, gestational age; GM, gray matter; HIP, hippocampus; HLHS, hypoplastic left heart syndrome; INS, insula; L, left; NAA, N-acetylaspartate; OCC, occipital; PAR, parietal; PCC, posterior cingulate cortex; PFC, prefrontal cortex; SES, socioeconomic status; SMG, supramarginal gyrus; SVP, single-ventricle physiology; TBV, total brain volume; TEMP, temporal; TGA, transposition of the great arteries; WM, white matter.

Fetal sonographic screening more often detects large brain malformations. Brain MRI studies during the third trimester or following birth achieve greater resolution. Fetal neurology consultations with interdisciplinary input offer opportunities for shared decisions with mothers and families, bounded by limits of imaging resolution at any time using current conventional testing modalities (59). Future clinical applications of research protocols will consider disease-specific mechanisms that reflect the impaired brain-placental axis using more sophisticated quantitative brain MRI technologies such as volumetrics and functional brain-placenta MRI imaging (Figures 2, 3) as early as during the second trimester (60, 61). Even prior to therapeutic interventions based on these novel diagnostic modalities, FNN involvement can better offer diagnostic and prognostic input regarding a more comprehensive group of MPF triad diseases. This involvement will strengthen continuity of care from fetal into postnatal life. Alternative interventions, more accurate prognostic predictions, and better-informed family counseling regarding future pregnancy planning will require shared clinical decisions among all stakeholders. Recent research contributions in fetal and neonatal neuropalliative care have demonstrated the importance of interdisciplinary collaborations using a comprehensive methodology of communication with families when challenging decisions are confronted (41, 62).

Fetal brain disorders often remain undetectable based on the current multiple levels of maternal surveillance (63). Many children with more subtle lesions may be missed with prenatal fetal surveillance even at more basic and tertiary obstetrical-pediatric centers. Preliminary studies are more often performed by less experienced fetal imaging personnel who are less likely to detect sonographic abnormalities at facilities that provide more limited maternal levels of care. These women are also less likely to be referred for interdisciplinary fetal neurology consultations based on geographic distance from a maternal-pediatric medical center even when sonographic abnormalities are detected. Neurologic disorders may elude prenatal detection until postnatal evaluations are offered for the neonate or child when communicable or non-communicable disease is present. Developmental delay or paroxysmal events before 2 years of age are the more common initial clinical phenotypic expressions by this “silent majority” (1). A more comprehensive evaluation throughout childhood requires consideration of reproductive and pregnancy contributions based on information provided to the pediatric neurologist. Childhood neuroimaging and neurogenetics studies as well as pediatric neurosurgery referrals are often later employed that reassess that disease pathways that occurred during the first 1,000 days with later G × E interactions are expressed across developmental time (64).

## Present biomarkers that distinguish MPF triad health from disease

Preconception exposures to diseases, medications, and environmental pollutants need to be addressed by women and their health providers to better formulate healthcare during each pregnancy (16, 65). Delaying planned pregnancies by effective methods of birth control is essential to address and reduce identified diseases or adversities for women that will affect their children. These priorities are particularly relevant to protect the adolescent female of child-bearing age who has increased biological and social risks that contribute to an abnormal pregnancy outcome (66). Reduction of specific risks can be attempted by optimizing nutrition, achieving effective weight loss, stopping or reducing prescribed medications that are potentially teratogenic, avoidance of harmful recreational substances, and treatment of medical including mental health conditions that elevate her stress responses.

These preconception choices need to be maintained throughout maternal care. Adverse effects from endogenous and exogenous toxic interplay may be activated or exaggerated during pregnancy that impair the woman’s multisystem readjustments during this and subsequent pregnancies (67). These adaptations also profoundly influence a woman’s mental and cognitive health now recognized within the field of matresence (68).

First trimester tests screen for genetic aneuploidy and carrier status, congenital infections, fetal anomalies, multisystemic maternal complications, and uteroplacental disease risks. Interventions may improve chances for more favorable outcomes, beginning with health maintenance that protects healthy reproductive and pregnancy exposomes (40).

Trimester-specific maternal testing helps monitor disease pathways that may be associated with fetal brain disorders despite no identified anomalies or destructive lesions on sonography. First trimester screening includes multiple non-invasive prenatal tests (69). Maternal blood samples utilize non-invasive plasma screening (NIPS) to detect for chromosomal abnormalities. Serum and urine studies assess for serologic evidence of pathogen-induced acute and infections (25). Placental function screening recommendations (70) assess for uteroplacental disease and immune dysregulation that contribute to adverse fetal outcomes such as miscarriage, fetal growth restriction, and prematurity. AFP screening and nuchal ridge measurements extend screening for NTDs and chromosomal syndromes. Amniocentesis and chorionic villus sampling are additional sources for maternal or placental sampling requiring mother’s consent.

Serial sonographic studies compare anthropometric ratios across three trimesters (71, 72) comparing somatic features to better detect anomalous or destructive changes. Increased disease severity may be represented by worsening phenotypic markers such as fetal growth restriction and hydrops fetalis. Functional assessments of fetal wellbeing apply fetal heart rate patterns with state reactivity, biophysical scales, and Doppler flow indices. These measures are compared with structural indices to offer more accurate predictions of the loss of fetal wellbeing with the possible association with fetal distress that carry increased disease risk. Testing choices throughout pregnancy are always guided by the mother’s accurate reportage of suspicious fetal movements and general health status. These historical and testing data help plan peripartum strategies to optimize labor and delivery and lessen neonatal complications.

Mother’s preconception and trimester-specific signs and symptoms will change relative to her adaptive or maladaptive responses across three trimesters of pregnancy (73). Screening results become more relevant during the third trimester as clinical signs of maternal diseases present or worsen such as hypertension, diabetes mellitus, and genitourinary infections. Non-inflammatory autoimmune disorders, pathogen-specific infectious diseases, and complex disorders such as diabetes and polycystic ovarian syndrome, blood group incompatibility, and genetic disorders also require closer scrutiny. Interdisciplinary planning using fetal interventions such fetal cardiac procedures for congenital heart lesions, fetal transfusions for blood group incompatibility and fetal hemorrhagic states, and amniotic fluid reductions for severe polyhydramnios may need to be pursued. Prenatal and exit surgical strategies, supported by evidence-based studies, help reduce postnatal mortality and morbidity. Specific conditions include procedures for complex congenital heart lesions, fetoscopic closure of open neural tube defects, placental vascular ablations to reduce the adverse effects from twin-to-twin transfusion syndrome, and removal of cystic hygromas following delivery to restore upper airway patency. These specialized procedures require expertise at specific obstetrical-pediatric centers with reliance on evidenced-based scientific findings. Hospitalizations for women experiencing acute medical problems such as systemic infections, hypertensive crises, diabetic ketoacidosis, or mental health crises may be lifesaving but are also potentially associated with worse outcomes for the MPF triad and child.

Placental and cord anomalies within sonographic limits of resolution may be documented as early as the first trimester. These findings may be correlated with MPF triad diseases with abnormal neurologic outcomes (74–76). Abnormal placental size, hemorrhage, implantation location as well as cord insertion, coiling, nuchal, or true knots present potential risks. These are potential markers of uteroplacental diseases that contribute to impaired fetal brain development (1). Multiple gestation pregnancies introduce concerns regarding placentation associated with placental vascular diseases such as twin-to-twin transfusion syndrome and fetal growth restriction. Large for gestational age weight status and concerns for uterine myometrial risks after primary or secondary rupture guide peripartum obstetrical choices for labor and delivery. These interventions depend on the status of maternal metabolic diseases such as diabetes mellitus and other medical conditions such as uterine anomalies and maternal obesity. Postnatal confirmation after gross and histological evaluations by the perinatal pathologist remains the gold standard when considering the effects by the placental exposome (77). International classifications describing four histopathological lesions have been correlated with neurologic disorders (78). G × E interactions resulting in TSI comprise the complicated disease pathways contributing to neurologic sequelae adversely which impair the neural exposome. Prenatal placental diagnostic modalities represent future opportunities to offer earlier interventions (79, 80).

Xenobiotic exposures potentially include thousands of potential pollutants with innumerable biological-chemical interactions that can affect the health status of the woman and maternal-placental-fetal triad, with effects across the lifespan (81, 82). Reports of urban and rural toxin exposures are published by federally regulated environmental agencies for public knowledge (83). However, no public health priorities yet systematically ensure that both persons and providers consistently find strategies to recognize and reduce exposures. High-risk resource-poor communities where medical deserts for maternal care prevail are particularly vulnerable. Untargeted and targeted screening for exogenous and endogenous toxins interacting with biological risks to the MPF triad are proposed (84, 85) but not yet clinically available to offer strategies that will reduce toxic exposures.

Barriers to appropriate levels of maternal care are also determined by attitudes and biases among providers, women, and their families that will impact quality of healthcare delivery. Effective cognitive clinical decisions by providers need to avoid errors of omission or commission by applying debiasing strategies (86). Numerous sources of bias are described which potentially hamper diagnostic accuracy and equity of healthcare delivery that adversely affects maternal and pediatric health (87).

Pediatric specialty consultations for high-risk MPF triads can potentially enhance healthcare perspectives. Prenatal pediatric care, however, continues to be challenged by the complexities of MPF triad diseases beyond traditional pediatric training programs to adequately prepare their trainees (46). Pediatric neurologists represent the subspecialty receiving the least amount of formal training as reported by this survey. Efforts to improve curriculum development require greater emphasis on maternal health before and during each pregnancy during neurology residency training to better prepare for career-long learning with relevance to promote brain health and reduce effects of disorders (1, 88). Pediatric healthcare providers representing all subspecialties require relevant FNN training that addresses biases and improves diagnostic accuracy.

Women need to be fully informed regarding their healthcare options regarding interventions. Providers are expected to share their medical knowledge and analysis calibrated to a woman’s cognitive abilities in a manner that is understandable and supportive. Reliance on misinformation by women based on faulty information from family members, friends, or social media diminishes trust in the healthcare system represented by their providers. Social determinants of healthcare continue to present challenges that reduce accurate and equitable clinical decisions given racial-ethnic, economic, geographic, and gender-identity barriers, even when providers perceive they have been offering equitable healthcare. Diversity and inclusion are required priorities by healthcare providers across all medical fields, particularly when serving children challenged with developmental disorders and associated neurologic morbidities (89).

## The diagnostic process applied to FNN clinical decisions

A two-step diagnostic approach guides FNN diagnostic decisions (1): (1)—A horizontal perspective identifies time-dependent phenotypic descriptions of neurologic signs and symptoms expressed by each woman, MPF triad, and neonate; (2)—a vertical perspective identifies relevant disease pathways that best explain phenotypic expression during that specific stage of developmental neuroplasticity. This horizontal-vertical diagnostic process helps maintain diagnostic accuracy as neurologic phenotypes appear across the lifespan.

Recognition of evolving G × E interactions specific to the prevailing clinical signs and symptoms represents expressions of a dynamic neural exposome that change across the developmental-aging continuum (90). Reproductive and pregnancy exposomes followed by pediatric exposomes represent the totality of exogeneous and endogenous toxic stressors before, during, and following each pregnancy that influence the expression of brain health or disease into adulthood (37). The case vignettes in this review emphasize how conditions associated with conception later influenced the developing MPF triad with effects on fetal brain structure and function expressed as sequelae during childhood into adulthood.

Diverse progenitor neuronal pathways are influenced by interrelated systems of the MPF triad beginning shortly at fertilization, even before the appearance of the primitive neural plate at conceptional days 17–19. The exposome concept helps elucidate developmental neuroplasticity processes within the developing nervous system across three trimesters that represent the fetal-neonatal brain connectome (91). Toxic stressor interplay (TSI) may adversely alter multisystemic MPF triad interactions affecting neurologic disease pathways affecting interrelated brain circuitries. Prenatal brain alterations will vary across three trimesters of pregnancy in location and severity. Exposome effects may be expressed by a minority of newborns as the “four great neonatal neurologic disorders, encephalopathy, seizures, stroke, and brain disorders of prematurity.” Childhood encounters with communicable and non-communicable diseases or adversities impair brain health expressed as neurologic disorders for the majority healthy neonatal survivors. An unknown percentage of these children were subjected to G × E interactions impairing the prenatal neural exposome without clinical expressions.

The diagnostic process must consider reproductive health of the woman prior to each pregnancy, followed by serial evaluations during the first 1,000 days of critical/sensitive neuroplasticity (1). Eighty percent of neuronal connectivity will be established by 2 years of age (92). Permanent neuroadaptive effects will later continue with life course phenotypic presentations particularly during adolescence and reproductive senescence. Adaptive or maladaptive responses impact women and their children, now recognized as the biological process of matrescence (68) during one or multiple pregnancies. Adversities reduce a woman’s health beyond her child-bearing years into old age with transgenerational consequences.

A dual cognitive process across disciplines was described over 50 years ago that facilitate the most accurate decisions (93). Fast intuitive thinking is often subconscious, automatic, and more susceptible to errors. This strategy accommodates to time-sensitive healthcare applications under acute and potentially lifesaving conditions such as medical emergencies, intensive care needs, and surgical procedures. Slow analytic thinking provides a supplemental method to reevaluate decisions with less time restraints and vulnerabilities from cognitive errors. Application of both processes helps minimize multiple biases to reach more accurate decisions with relevance across multiple healthcare disciplines (94, 95). This dual cognitive process has important applications for interdisciplinary FNN training and career-long practice. Integrating primary and subspecialty healthcare approaches will more likely achieve useful knowledge transfer among all stakeholders across maturational time during and following pregnancy. This strategy when applied by both patients and providers will more likely achieve more accurate decisions regarding brain health throughout childhood into adulthood.

## The science of uncertainty applicable to FNN

Since future relevant information cannot be anticipated during a specific clinical encounter, the science of uncertainty is applicable over serial reassessments, recognizing the uncertainties inherent using the scientific method. A recent scoping review suggests a four-step analytic approach (96) that can assist with FNN clinical decisions: (1) recognition of uncertainty for any clinical situation over developmental time is the required first step, focused for this discussion on the woman, MPF triad, neonate, or child. (2) Classification of uncertainty in terms of source, issue, and the specific stakeholder is required. Probability, complexity, and ambiguity are three sources of uncertainty surrounding outcomes and interventions. Uncertainty presents when the probability of the diagnostic choice or treatment is neither high nor low given the available evidence presented by maternal, MPF triad, or pediatric clinical information. Complexity influences uncertainty given multiple G × E interactions across developmental time that contribute to changing phenotypic expressions by the woman, MPF triad, or child. Ambiguity represents two interacting quandaries: (a) a lack of evidence based on subclinical status of a disease pathway using limited prenatal or postnatal surveillance testing options as reliable biomarkers and (b) differing opinions among consultants regarding their specific frames of reference applied to diagnostic and/or therapeutic choices using available data. Classifying uncertainty also depends on identifying the issues valued by each stakeholder. Relevance to scientific principles, practical healthcare situations or personal beliefs of the patient and family must be recognized. The level of uncertainty with each prenatal or postnatal clinical decision needs to determine which party assumes the role as primary decision maker at that time.

Clinicians’ abilities to consider opinions of key stakeholders influence effective management of uncertainty regarding FNN decisions during pregnancy planning, prenatal surveillance, and postnatal healthcare encounters. Recognition of differing viewpoints provides an opportunity to identify conflict and negotiate a consensus. This shared mind set incorporates patient preferences into action plans with goal setting as prerequisites for future shared decision-making. Repeated interactions among patients, families, and clinicians will later introduce new information that will require revised clinical decisions that impact health of the woman, MPF triad, and child.

Knowledge acquisition by the clinician practicing FNN first requires effective education to integrate available evidence and offer time-dependent clinical decisions. Horizontal-vertical diagnostic approaches to cognitive thinking are optimally established during formal FNN training (1). Career-long experiences reinforce this earlier teaching to maintain accurate decisions, strengthened by insight and judgment that recognizes shared decision-making. Avoidance of bias must be explicitly taught and subsequently reinforced through career practice to successfully achieve effective healthcare delivery and scientific research endeavors.

The clinician’s overall approach to clinical decisions-making merges four strategies involving intuition, protocol-driven, team-based, and shared actions with patients and families. Collective assessments incorporating synthesis and reflection guide the clinician practicing FNN to apply newly acquired information to complex clinical decisions for the woman, MPF triad, and child across neurodevelopmental time. Best approaches to manage uncertainty need to incorporate bioethical priorities into clinical decisions specific to each patient and their family. FNN curriculum must introduce bedrock principles of cognitive processing that recognize humanistic clinical decisions that value moral choices. Teaching of uncertainty to the trainee must maintain bio-social equipoise across one’s professional lifetime of practice. Continuing education for providers reinforced after repeated clinical encounters can help prioritize social determinants of health when delivering equitable person-centered healthcare. Those persons receiving healthcare also require an understanding of the science, so they can contribute to their wellbeing limited by the uncertainty of science.

## Lessons learned from the three case vignettes regarding shared decisions

Each mother-child dyad in this review highlights opportunities and challenges involved with shared clinical decisions between the woman and her healthcare providers that rely on a shared fund of knowledge. Trust by the woman, her partner, and family relies on the accuracy of a proposed diagnosis and recommendations dependent on the conveyed information at that encounter. With each case vignette, informed clinical decisions were reached based on the science of uncertainty to ascertain the optimal diagnosis given available information at a specified time during pregnancy. Choices also required that the woman and her providers acknowledge the uncertainty of science-based statistical probabilities confined by present day diagnostic limitations. Group predictions will not always apply to person-specific experiences.

The woman in the first case study considered her own internet-based research regarding her child’s presumed fetal brain malformation which prompted seeking a second opinion. This medical advice and counsel required an informed shared clinical decision that was time-dependent. Legal constraints by state law prohibited an elective pregnancy termination beyond a specified gestational age. Her participation in this re-evaluation employed a “slow thinking” cognitive approach with her new providers within this arbitrary time restraint. A diagnosis of an isolated meningocele was based on a fetal brain MRI interpretation, again supported by a repeat normal serum AFP result. More favorable or normal maternal and fetal outcomes were discussed, and mother chose to continue her pregnancy.

Mother’s subsequent third trimester hypertensive disorder diagnosed as severe preeclampsia required important time-dependent clinical decisions given new risks for adverse maternal and fetal outcomes. Ischemic placental disease (97, 98) can be associated with hypertensive disorders of pregnancy which carry increased risks for her daughter’s preterm birth as well as maternal mortality and morbidity. Peripartum and neonatal complications consequently increase risks for neurologic sequelae for her child (99). Risks for increased neurologic morbidities were based on complications from encephalopathy of prematurity that combined prenatal and postnatal factors (100). Cerebral dysgenesis remained an earlier identified risk, despite the presumed meningocele given limitations in fetal brain MRI resolution. Childhood assessments were performed by multiple specialties in addition to pediatric neurology and neurosurgery, anticipating subsequent need for ongoing medical and educational interventions. This child fortunately resolved her earlier developmental deficits with a normal neurologic outcome into the third decade.

The second case vignette illustrated divergent choices by two women given their decisions not to obtain a fetal brain MRI to more accurately consider the prenatal diagnosis of TSC complex. Most children with multiple fetal cardiac rhabdomyoma have a higher probability of the genetic diagnosis of TSC (9). Prenatal brain MRI offers earlier information to consider this prenatal genetic diagnosis. Medical and ethical challenges follow regarding continuation or termination of a pregnancy. A postnatal brain MRI will be required for greater accuracy. The first woman’s choice not to pursue a fetal brain MRI was based on her unilateral internet-based research that rejected the medical advice at her first medical center to pursue further evaluation. She assumed a poor outcome which represented premature closure bias despite medical advice that suggested the need for additional testing. As with the first case vignette, there were legal restraints that required out-of-state medical intervention for pregnancy termination before a specific gestational age. After her elective termination, no postmortem assessment eliminated pathological documentation of TSC. Future pregnancy planning was not possible without this information, particularly since neither parent had a positive family history nor exhibited clinical signs of TSC that would have suggested autosomal dominant genetic inheritance.

The other woman in this second case vignette also chose not to obtain a prenatal brain MRI and requested pregnancy care followed by a postnatal evaluation for TSC. This presented a choice by her health providers to respect her value-based decision based on her beliefs (101). She and her partner supported by their families were committed to offer their child a meaningful life despite her child’s unknown life expectancy and quality of life assuming the diagnosis of TSC. Mother’s personal value system transcended strict medical concerns for neurologic sequelae associated with TSC, reaching a moral decision based on her religious convictions communicated to her healthcare providers. Avoidance of eugenic-based decisions to end a pregnancy presents ethical challenges, given the uncertainty of reaching inaccurate genetic diagnoses despite advanced testing that will more likely predict a suboptimal outcome (102). This woman’s decision differed from the other woman’s choice to terminate her pregnancy, desiring to maintain her pregnancy despite a probable poor outcome. She chose to delay further testing until after birth without considering additional prenatal diagnostic information. Medical uncertainty regarding outcome must respect family-valued shared decisions despite maternal and pediatric risks associated with these decisions. It also remains essential to avoid healthcare disparities that will prevent or impede equitable maternal healthcare delivery. Public health policies require respect for the effects of social determinants on health. Such policies help avoid institutional biases against women and their partners based on race-ethnicity, socioeconomic circumstances, or geographic distance which will reduce their ability to obtain equitable healthcare and quality of life for themselves as well as for their special needs children (89).

The mother in the last case vignette presented an alternative scenario with challenges to prenatal clinical decisions. Her unilateral internet-based information was biased by inconclusive scientific evidence that was compounded by recommendations at the institution where her unborn child received a fetal spina bifida repair. Fetoscopic closure of her child’s open neural tube defect ignored scientific uncertainties amplified by biases by the specialists who offered this intervention. Mother’s personal motivation to pursue an untested prenatal surgical procedure at her own expense was reinforced by an out-of-state healthcare system that claimed surgical expertise that would improve outcome with inconclusive peer-reviewed medical evidence regarding efficacy. The subsequent MOMS reported fewer complications from shunt-dependent hydrocephalus and greater ambulatory abilities as this chosen institution claimed. However, the ethical decisions integrated into the MOMS randomization for surgery excluded children with genetic disorders who would be at greater risk for neurodevelopmental outcomes despite this intervention. This child’s AMC combined with his extensive neural tube defect would have made him ineligible for the procedure.

Parents seeking the best outcome for their unborn child are vulnerable to biased medical information acquired from the internet that lack evidence-based research to justify an elective intervention, such as prenatal closure of an open neural tube defect. The MOMS findings established a more bioethically sound basis for shared clinical decisions that weigh the possible reduction of specific childhood morbidities against a child’s person-specific risk factors such as this child’s AMC. While the child received compassionate care into adulthood, the parents’ decision to decline future pregnancy planning followed by their later divorce was negative outcomes that reduced their quality of life both as individuals and as parents. Healthcare providers have a responsibility to provide to the woman and her partner an understandable summary of the known and unknown features to reach an accurate diagnosis to plan future pregnancies.

Current scientific advances continue to present challenges affecting medical choices for women, their families, and their healthcare providers. Prenatal genetic testing of genetic carrier status for specific diseases can now identify TSC 1 or 2 missense variants (103) or complex allelic abnormalities linking a NTD with AMC (104) based on whole-exome sequencing or high through-put genomic sequencing. Prenatal drug treatments provisionally suggest that prenatal sirolimus treatment can reduce the size of cardiac rhabdomyomas associated with TSC, with unclear benefits that might reduce the number and size of brain hamartomas (105). Research-based second trimester quantitative brain-placental imaging similarly offers opportunities and challenges regarding the “correct decision.” Therapeutic medical and surgical intervention trials need to be conducted to establish reasonable scientific benefit/risk ratio analyses regarding survival and an improved quality of life. These future technologies must also consider the complex bioethical decisions by women depending on their personal values and beliefs. Healthcare providers must continue to participate in shared clinical decisions. The science of uncertainty together with the uncertainty of science will continue to challenge diagnostic choices and therapeutic interventions at critical periods of neuroplasticity beginning with conception. Preconception testing may better inform women and their partners for optimal pregnancy planning as novel genetic tests are developed. There does not, however, exist a population-based genetic screening program with universal access to women without limitations based on healthcare disparities. Even if such a program existed, implementation would be seriously hampered given the persistently high percentage of unplanned pregnancies without access to reproductive health as reported in both resource-rich and poor countries (106).

These limitations are further compounded by women who live at greater geographic distances from institutions that have the required interdisciplinary expertise that include medical and surgical pediatric subspecialties. Pediatric neurology and neurosurgery opinions require interdisciplinary collaborations among obstetrical, neonatal, and pediatric system-specific healthcare providers, preferably a maternal-pediatric health center. These teams also need to provide professional input for mental healthcare by providers in psychiatry and psychology. These consultations must be aligned with nursing and social work collaborations as shared decisions are reached with women, their partners, and families that consider values and beliefs as well as scientific facts.

## TSI worsens all stressors complicating shared clinical decisions

Genetic diagnostic advances will certainly improve but cannot predict every pregnancy complication affecting the MPF triad from acquired diseases or adversities that can contribute to an impaired neural exposome from abnormal reproductive and pregnancy exposomes (39). The prognostic unknowns were exemplified by the woman in the first case vignette who experienced third trimester severe preeclampsia. Despite earlier identification of an isolated meningocele, she and her child were later challenged by medical complications as a MPF triad from ischemic placental disease associated with preeclampsia and prematurity. Prenatal and postnatal consequences of encephalopathy of prematurity increased her child’s risks for neurologic sequelae. Unexpected postnatal complications from TSI as for the child will always challenge the abilities of healthcare providers to reach constructive and ethically sound shared decisions. Difficult medical choices need to be reached in the outpatient, inpatient, or intensive care settings when communicable or non-communicable diseases further compromise a child’s brain health with increasing age.

Preconception and pregnancy mental health of the woman and her partner influence fetal brain development with childhood structural and functional effects (107, 108). Pre-existing mental health disorders of the woman or her partner such as from depression and anxiety can worsen stressor effects during pregnancy (109). Situational psychological stressors are experienced by parents struggling with difficult medical decisions even without preexisting mental health disease. Adverse effects from stress later threaten their child’s mental health with adult consequences during their own reproductive years (110). Post-translational disease pathways include epigenetic mechanisms such as methylation as well as mosaicism, somatic variation, and imprinting, Different post-translational mechanisms result in maladaptive stressor responses through TSI that are expressed across each and successive generations (111). Excessive release of endogenous corticosteroids and noradrenergic substances contributes to multiple forms of altered genetic expression. This results from the woman’s abnormal hypothalamic-pituitary axis function with and without underlying mental health disorders. Recreational and prescribed medications add xenobiotic exposures that worsen these adverse effects on fetal brain development (112, 113).

Preconception and pregnancy medical care optimally require systematic investigations of maternal stress using validated questionnaires such as standard depression screening tests, starting with the woman’s first prenatal visit (114). Adverse childhood experiences by the woman and her partner long before planned or unplanned pregnancies need to be identified. Continuity of risk increases during each pregnancy, given the challenges of physiological adaptation experienced by the woman (115) as well as the psychological adjustments required by both parents (116). Suboptimal parenting more likely leads to adverse childhood experiences expressed as neurodevelopmental impairment, beginning during the first 1,000 days with life course effects (117, 118). Mental health interventions may require pharmacological management combined with group or individual behavioral supports.

## Balancing a brain health strategy with compassionate care

Women’s and children’s goals for lifelong brain health (119) help promote a brain capital strategy through the science of social-to-biological transitions (120). Present brain research, innovation, regulatory, and funding systems are unfortunately siloed, creating boundaries in our understanding of the dynamic neural exposome that require interdisciplinary perspectives that highlight healthcare for women and children. Concepts involving the developmental-aging paradigm need a life course approach that merges neurologic-mental health priorities with multisystemic perspectives within a social context (42). Pediatric neurologists will better contribute to a brain health capital strategy by incorporating FNN training to career-long learning as their patient experiences increase (39).

Maternal-child health initiatives need to integrate reproductive, pregnancy, and pediatric priorities to achieve effective healthcare that improves brain capital across the lifespan (121). The United Nations Agenda for Sustainable Development presented 17 sustainable development goals (SDGs) at the end of the 20th century as a blueprint for global peace and prosperity. Yearly updates report on continued attempts (122) to achieve meaningful improvements by 2030. These strategies suggest public health programs that can improve health and education, reduce inequality, spur economic growth, and reduce the adverse effects of climate change with lifelong brain health priorities (123).

**Figure 4:**
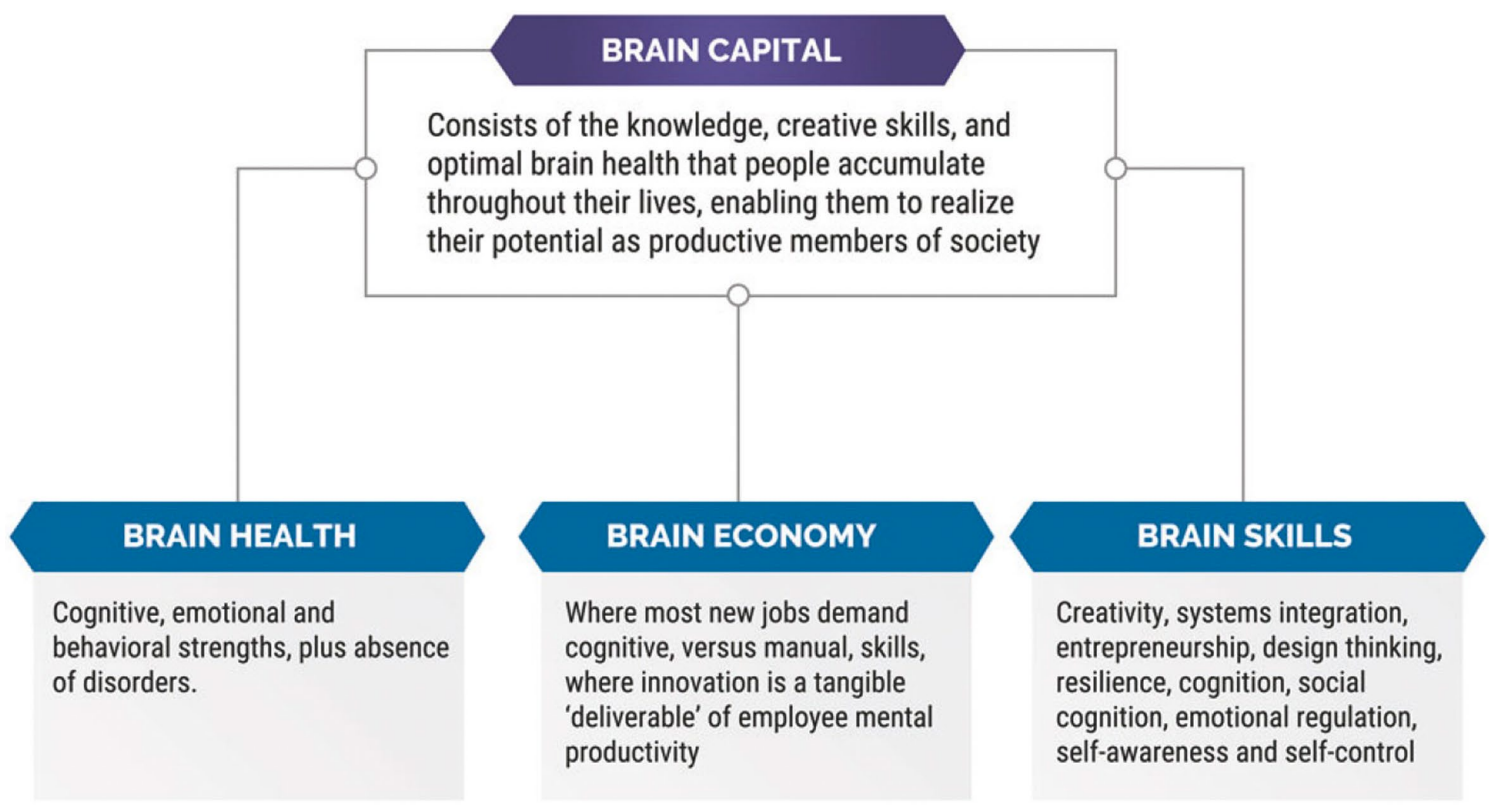
This figure provides the definition of a brain health capital strategy, as well as explanations of three sub-components that comprise brain capital, i.e., brain health, brain economy, and brain skills (42).

Polycrisis events exemplified by more recent COVID and opioid pandemics further affected by ongoing worldwide armed conflicts reaffirm the need for healthcare policies that protect vulnerable women and children exposed to the most extreme adversities (124). Synergy among disciplines stresses sustainability of brain health across each lifespan through shared learning about the neural exposome by healthcare providers and all stakeholders. A science-based agenda for life course health policy can potentially mitigate TSI effects and preserve brain health for successive generations (83, 125). This brain health capital strategy can only be realized within a society that prioritizes government programs that recognize all persons’ rights for optimal health and wellbeing beginning with women and children (Figure 4).

Interdisciplinary FNN training helps promote person-centered clinical and research collaborations between neurologists and other healthcare disciplines. Multi-institutional protocols need to explore novel diagnostic and therapeutic strategies that will benefit women, children, and families relative to life course TSI through reproductive senescence. Age-specific diagnostic modalities choose genotypic-phenotypic biomarkers that will help promote effective neurotherapeutic interventions, particularly during critical-sensitive time periods of neuroplasticity across each lifespan (126).

## Author contributions

MSS: Writing – original draft.
